# Comparative Efficacy and Safety of Vasodilators Used to Prevent Internal Thoracic Artery Spasm in Coronary Artery Bypass Grafting: A Systematic Review

**DOI:** 10.7759/cureus.100466

**Published:** 2025-12-31

**Authors:** Muhammad Qaiser Aziz khan, Shahzad Ahmad, Manahil Awan, Zeshan Navid Syed, Shashwat Shetty

**Affiliations:** 1 Cardiac Surgery, Liaquat National Hospital, Karachi, PAK; 2 Cardiac Surgery, Liaquat National Hospital, karachi, PAK; 3 Cardiac Surgery, Blackpool Teaching Hospital National Health Service (NHS) Trust, Lancashire, GBR; 4 Orthopaedics, Hillingdon Hospital, Uxbridge, GBR

**Keywords:** cabg, glyceryl trinitrate-verapamil, internal thoracic artery spasm, papaverine, vasodilators

## Abstract

Internal thoracic artery (ITA) spasm during coronary artery bypass grafting (CABG) can compromise graft flow and perioperative outcomes. This review systematically evaluates the comparative efficacy and safety of vasodilators used to prevent ITA spasm, including papaverine, glyceryl trinitrate-verapamil (GV) solution, systemic diltiazem, and efonidipine. Six studies comprising clinical, ex vivo, and in vitro investigations were included. GV solution consistently demonstrated superior vasodilatory efficacy, rapid onset (2.9 min), and high potency, achieving near-complete ITA relaxation and increasing intraoperative free flow by up to 95%, with minimal adverse events. Papaverine effectively prevented spasm at high concentrations but showed a slower onset. Systemic diltiazem improved graft flow safely, while efonidipine exhibited dose-dependent relaxation in ex vivo models, targeting both L- and T-type calcium channels. Delivery method influenced efficacy, with topical or intraluminal administration providing rapid, direct vasodilation. Overall, combination therapy (GV solution) offers a practical and safe approach for intraoperative ITA spasm prevention.

## Introduction and background

The internal thoracic artery (ITA), also known as the internal mammary artery, supplies the chest wall and typically divides into the musculophrenic and superior epigastric arteries at the level of the sixth or seventh intercostal cartilage [1]. Histomorphometrically, the ITA is composed of three main layers: the tunica intima, the tunica media, and the tunica adventitia. The tunica intima is externally limited by a well-defined internal elastic lamina (IEL) and includes the endothelium, a crucial component in cardiovascular homeostasis and vascular regulation. The tunica media, the thickest vascular layer, contains circumferentially aligned elastic lamellae and vascular smooth muscle cells (VSMCs) situated between the internal and external elastic laminae. Although initially classified as an elastic artery, the ITA exhibits a varying histological pattern along its course from the subclavian artery to the epigastric bifurcation, gradually shifting in the proportion of elastic and muscular elements and transitioning from a more elastic artery proximally to a more muscular artery distally [2]. These structural and functional characteristics contribute to its exceptional durability, resistance to atherosclerosis, and long-term patency, which is why the ITA is widely used as the preferred conduit in coronary artery bypass grafting (CABG).

ITA remains the conduit of choice for coronary artery bypass grafting (CABG), offering superior long-term patency exceeding 90% at 10 years, compared with 50-60% for saphenous vein grafts [3]. The left ITA (LITA) in particular demonstrates excellent resistance to atherosclerosis and improved survival benefits when used to revascularize the left anterior descending artery. Despite these advantages, perioperative ITA spasm continues to pose a clinically significant challenge, occurring in 0.4% of CABG procedures [4]. Spasm can result in an abrupt reduction in graft flow, hemodynamic instability, perioperative myocardial ischemia, and, in severe cases, early graft failure. Vasodilator therapy plays a central role in preventing and treating ITA spasm. Multiple pharmacologic agents have been used, including papaverine, glyceryl trinitrate (GTN), calcium channel blockers (CCBs), and combination solutions such as glyceryl trinitrate-verapamil (GV). These agents differ markedly in their mechanisms of action: papaverine acts through phosphodiesterase inhibition, nitrates via nitric oxide signaling, and CBS through L- or T-type channel blockade [5].

Recent experimental and clinical evidence suggests that certain regimens, such as GV solution, may provide faster and more complete vasodilation, with studies reporting relaxation rates of 95-100% compared with 50-60% for papaverine under similar conditions [6]. This variability highlights the need for a structured evaluation of vasodilator efficacy and safety. However, current evidence spans heterogeneous study designs, including clinical trials, non-randomized studies, in vitro organ bath experiments, and ex vivo vascular pharmacology. These methodological differences complicate direct comparisons and have prevented consensus regarding the optimal vasodilator. Additionally, newer agents such as efonidipine, which block both L- and T-type calcium channels, have shown promising effects demonstrating dose-dependent relaxation and suppression of vasoconstrictor-induced tension by 40-70% in recent experiments, but have not been evaluated in a unified evidence synthesis [7]. The lack of consolidated, comparative data limits clinical decision-making during CABG, where vasodilator choice affects both intraoperative stability and long-term graft performance.

The primary aim of this review is to systematically compare the efficacy of vasodilators used to prevent ITA spasm in CABG across clinical, in vitro, and ex vivo studies. The secondary aims are to evaluate the safety profiles of these vasodilators, compare their mechanisms of action and onset times, and identify the concentrations, delivery methods (topical, intraluminal, systemic), and regimens associated with optimal graft flow. By integrating quantitative and qualitative findings, this review seeks to provide evidence-based recommendations to optimize ITA preparation and reduce perioperative spasm incidence.

## Review

Materials and methods

Search Strategy

A systematic search was conducted across PubMed, Embase, Scopus, and the Cochrane Library from inception to November 2025 following PRISMA guidelines 2020 [8]. Search terms included combinations of “internal thoracic artery,” “mammary artery,” “spasm,” “vasodilator,” “papaverine,” “glyceryl trinitrate,” “verapamil,” “calcium channel blocker,” and “coronary artery bypass.” Boolean operators and MeSH/Emtree headings were used to maximize sensitivity. Additional studies were identified through reference-list screening and citation tracking. Search results were imported into a reference manager, and duplicates were removed before screening. The PRISMA flow process included identification, screening, eligibility assessment, and inclusion.

Eligibility Criteria

Eligibility criteria were developed using the PICO framework to ensure appropriate and consistent study inclusion [9]. The study population included adult patients undergoing coronary artery bypass grafting (CABG) using internal thoracic artery (ITA) grafts, as well as studies using human ITA or left internal mammary artery (LIMA) segments in ex vivo or in vitro experimental models. The Intervention comprised any vasodilator administered to prevent or attenuate ITA spasm, including papaverine, glyceryl trinitrate, verapamil, glyceryl trinitrate-verapamil (GV) solution, and calcium channel blockers such as diltiazem or efonidipine. Acceptable comparators were placebo, no treatment, baseline measurements, vasoconstrictor challenge, or alternative vasodilators used in clinical or laboratory settings. Studies were eligible only if they directly evaluated in situ internal thoracic artery (ITA) vasoreactivity or flow following topical or intraluminal application of vasodilator solutions under standardized intraoperative, ex vivo, or in vitro conditions. Randomized trials primarily evaluating systemic pharmacologic strategies, postoperative clinical outcomes, or indirect markers of vasospasm without direct ITA assessment were excluded. 

Eligible outcomes included measures of ITA relaxation, graft free flow, tension reduction, spasm suppression, onset time, molecular effects, and safety profiles. Studies were included if they were randomized controlled trials, non-randomized clinical trials, ex vivo studies using human ITA tissue, or in vitro organ bath experiments assessing vasodilatory responses, and if they provided full-text, English-language data reporting at least one ITA-related vasodilatory or antispastic outcome. Studies were excluded if they involved animals, were case reports or small case series, editorials, narrative reviews, or conference abstracts lacking complete data, or if they evaluated non-ITA conduits without ITA-specific findings. This framework ensured that all included studies directly addressed vasodilator efficacy or safety in preventing ITA spasm and matched the methodological scope of the review.

Study Selection

All records identified from the database search were imported into EndNote™ for deduplication. Titles and abstracts were screened independently by two reviewers. Full-text articles were retrieved when inclusion could not be determined from the abstract alone. Discrepancies were resolved by consensus or by consultation with a third reviewer. The final study set included both clinical and experimental studies directly evaluating vasodilators in human ITA tissue or during CABG procedures.

Data Extraction

A standardized extraction form captured study characteristics (year, design, sample size, ITA preparation method), population/tissue source, vasodilator agent(s), concentrations/dosages, delivery modality (topical, intraluminal, systemic, organ bath), comparators, primary and secondary outcomes, onset time, molecular data (where available), and safety events. For in vitro/ex vivo studies, additional fields captured vasoconstrictor type, baseline tension, and percentage relaxation. Extraction was performed independently by two reviewers to minimize transcription errors and bias.

Risk of Bias Assessment

Risk of bias was assessed using tools matched to each study design. Clinical trials assessed with the RoB-2 tool demonstrated variable quality, with some showing moderate risk due to insufficient reporting of randomization procedures, limited allocation concealment, and lack of blinding. However, the use of objective flow measurements helped reduce detection bias [10]. Non-randomized clinical research evaluated using ROBINS-I showed a moderate risk, primarily related to potential confounding arising from non-random allocation despite complete and objective outcome assessment [11]. In contrast, all laboratory investigations, including in vitro, ex vivo, and molecular studies, were appraised using the NIH Quality Assessment Tool for Before and After Studies Without Control Groups and were consistently rated as low risk, reflecting controlled experimental conditions, standardized methodologies, reproducible protocols, and objective tension measurement techniques [12].

Data Synthesis

Given the considerable heterogeneity in study designs, vasodilator types, concentrations, delivery methods, and outcome measures, quantitative pooling through meta-analysis was not feasible. Therefore, a structured narrative synthesis was undertaken. Studies were grouped into three main categories: clinical free-flow assessments during CABG, ex vivo or in vitro experiments evaluating vasodilatory efficacy, and mechanistic or molecular investigations. Findings across these categories were integrated to enable comparison of vasodilators with respect to the magnitude and speed of relaxation achieved, the consistency of their effects across different experimental and clinical contexts, and their overall safety profiles.

Registration

This review was not registered in the PROSPERO international prospective systematic review database. Although PROSPERO registration enhances transparency and reduces the risk of reporting bias, the methodological steps of this review, including search strategy development, study selection, data extraction, and risk of bias assessment, were predefined and conducted in accordance with Preferred Reporting Items for Systematic reviews and Meta-Analyses (PRISMA) 2020 guidelines.

Results

Study Selection Process

**Figure 1:**
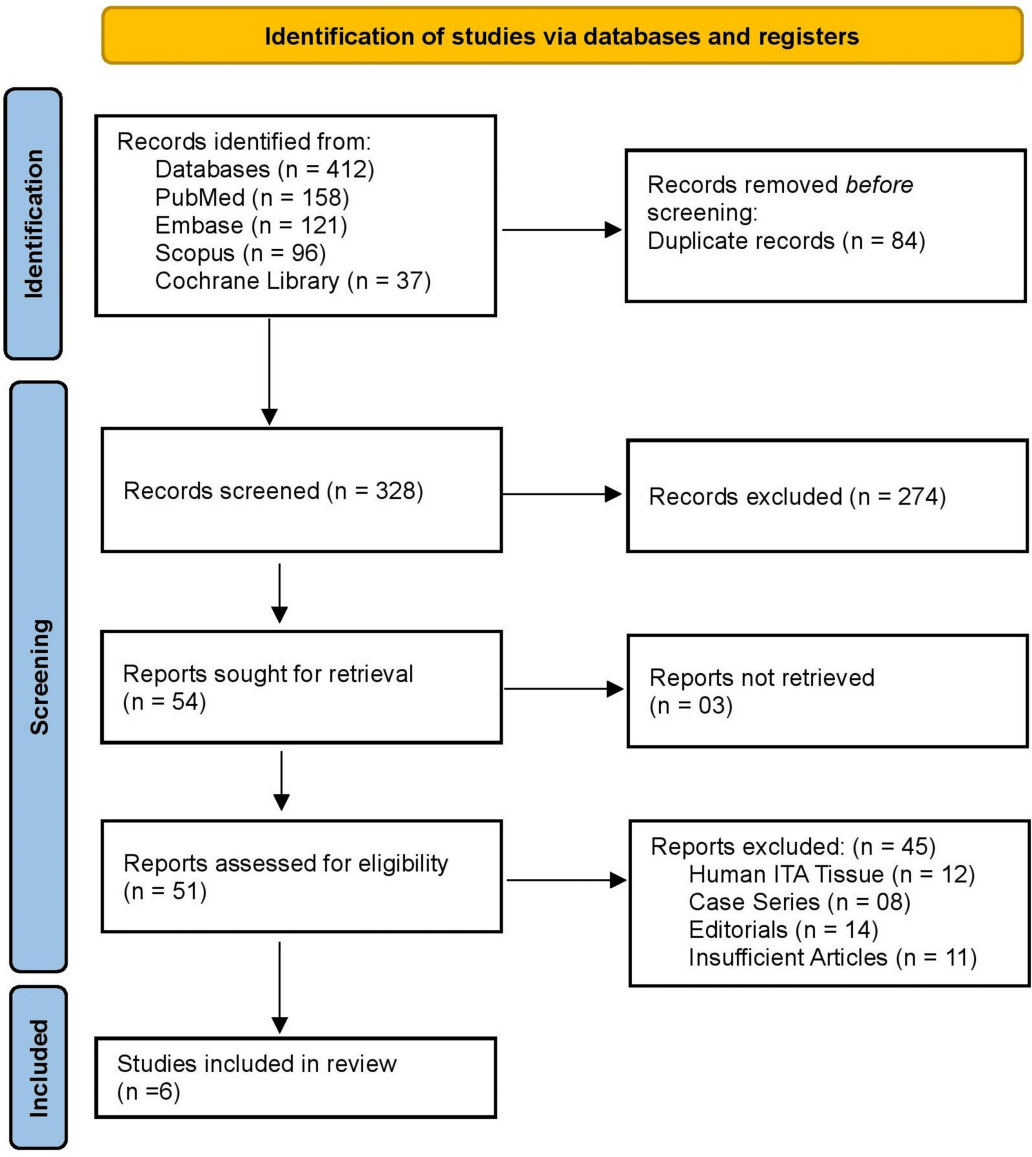
PRISMA 2020 flow diagram PRISMA: Preferred Reporting Items for Systematic reviews and Meta-Analyses.

Figure 1 shows a total of 412 records were identified through database searching (PubMed 158, Embase 121, Scopus 96, and the Cochrane Library 37). After removing 84 duplicates, 328 records remained for title and abstract screening, of which 274 were excluded. Because they did not meet the predefined eligibility criteria. The most common reasons included: studies not evaluating the internal thoracic artery (e.g., focusing on other grafts or vascular beds); studies assessing systemic pharmacologic strategies rather than topical or intraluminal vasodilator solutions; absence of direct assessment of ITA vasoreactivity, spasm, or flow; non-comparative designs (reviews, editorials, case reports); animal-only studies without translational relevance; and conference abstracts without full-text availability. Full texts were sought for 54 reports; three were not retrievable, leaving 51 studies for eligibility assessment. Of these, 45 were excluded based on predefined criteria: 12 were animal studies that did not involve human ITA tissue, eight were case reports or very small case series, 14 were editorials or narrative reviews lacking original data, and 11 were conference abstracts without sufficient methodological detail. Ultimately, six studies met all inclusion criteria and were included in the review.

Characteristics of the Selected Studies

Table 1 shows six studies evaluating the efficacy of various vasodilators in preventing internal thoracic artery (ITA) spasm. In clinical settings, intraluminal or topical glyceryl trinitrate-verapamil (GV) solution significantly increased free flow in 31 patients undergoing ITA grafting, with flow rising approximately 95% compared to 53% in controls, and no major complications were reported, indicating effective spasm prevention [13]. In vitro studies using 14 human left internal mammary artery (LIMA) segments showed that GV solution produced 100% relaxation with a faster onset (2.9 vs 8.1 min) and higher potency (pIC50 6.54 vs 4.58) compared with papaverine, effectively abolishing induced spasm [14]. Papaverine alone completely blocked vasoconstriction at 100 µM in six human ITA segments, establishing its effective dose for spasm prevention [15]. Systemic diltiazem infusion in 60 patients undergoing coronary artery bypass grafting (CABG) resulted in higher first and second free flow measurements (53.8 ± 30.1 and 72.3 ± 35.4 mL/min) compared with systemic nitroglycerin (25.7 ± 16.2 and 48.9 ± 23.8 mL/min), demonstrating a significant reduction in perioperative ITA spasm while remaining hemodynamically well tolerated [16]. Efonidipine, a dual L- and T-type calcium channel blocker, produced dose-dependent relaxation and inhibited KCl- and U46619-induced contraction in 54 discarded human internal mammary artery (IMA) segments, with molecular evidence showing downregulation of Cav1.2 and Cav3.1 channels [17]. In off-pump CABG, the GV solution also demonstrated superior efficacy over papaverine in 60 patients, significantly increasing free-flow and preventing ITA spasm, with no deaths reported in the GV group compared with two in the papaverine group [18].

**Table 1:** Characteristics of the selected studies ITA: internal thoracic artery, LIMA: left internal mammary artery, IMA: internal mammary artery, CABG: coronary artery bypass grafting, GV: glyceryl trinitrate-verapamil, CCB: calcium channel blocker, KCl: potassium chloride.

Authors & Year	Population (P) / Tissue	Intervention (I) / Vasodilator or Technique	Comparator (C)	Outcome (O)	Effect on ITA	Observations	Spasm
He et al., 1994 [13]	31 patients undergoing ITA grafting	Intraluminal/topical GV solution (GTN + Verapamil)	Ringer’s solution / no treatment	Free-flow (ml/min)	Flow increased ~95% in GV vs ~53% in control	Safe, no major complications	Significantly reduced ITA spasm in the GV group
Shafa et al., 2017 [14]	14 human LIMA segments	GV solution in the organ bath	Papaverine solution	Relaxation %/onset time	100% relaxation faster; pIC50 6.54 vs 4.58; onset 2.9 vs 8.1 min	In vitro study, high reproducibility	The GV solution abolished induced spasm
Tanaka-Totoribe et al., 2005 [15]	6 human ITA segments	Papaverine at various concentrations	Vasoconstrictors	Vessel contraction (%)	Completely blocked vasoconstriction at 100 µM	Ex vivo tissue; effective dose established	Prevented all induced spasm
Tabel et al., 2002 [16]	60 patients undergoing CABG	Systemic Diltiazem infusion	Systemic Nitroglycerin	First & second free flow (ml/min)	Diltiazem: 53.8 ± 30.1 & 72.3 ± 35.4 vs NTG: 25.7 ± 16.2 & 48.9 ± 23.8	Safe; hemodynamically well-tolerated	Diltiazem significantly reduced perioperative ITA spasm
Yin et al., 2023 [17]	54 discarded human IMA segments	Efonidipine (L- and T-type CCB)	Mibefradil/baseline	Vessel tension reduction (%)	Dose-dependent relaxation; inhibited KCl- & U46619-induced contraction	Molecular evidence: downregulation of Cav1.2 & Cav3.1	Potent prevention of experimentally induced spasm
Akter et al., 2024 [18]	60 patients undergoing off-pump CABG	GV solution	Papaverine	Free-flow (ml/min)	Significantly higher in GV vs Papaverine	No deaths in the GV group; two in papaverine	The GV solution prevented ITA spasm more effectively

Risk of Bias Assessment

Table 2 summarizes the risk of bias across the included studies. He et al. (1994) showed a moderate risk due to unclear randomization and lack of allocation concealment, though objective flow measurements reduced detection bias [13]. Shafa et al. (2017) demonstrated low risk, with standardized in vitro protocols ensuring reproducibility [14]. Tanaka-Totoribe et al. (2005) also showed low risk owing to controlled ex vivo conditions and precise dose-response procedures [15]. The randomized controlled trial by Tabel et al. (2002) exhibited low risk with adequate randomization, objective outcomes, and complete reporting [16]. Yin et al. (2023) maintained low risk through rigorous laboratory methods and molecular validation [17]. Finally, Akter et al. (2024) presented a moderate risk due to non-random allocation and potential confounding, although objective outcome assessment minimized additional bias [18].

**Table 2:** Risk of bias assessment RCT: randomized controlled trial, ITA: internal thoracic artery, CCB: calcium channel blocker, NIH: National Institutes of Health, RoB-2: Risk of Bias tool for randomized trials, ROBINS-I: Risk of Bias in Non-randomized Studies of Interventions.

Study	Design	Risk of Bias Tool	Rating	Justification
He et al., 1994 [13]	Clinical trial	RoB-2	Moderate	Randomization and allocation concealment were not clearly described, increasing selection bias risk. Outcome assessors were not reported as blinded, though objective free-flow measurements reduced detection bias.
Shafa et al., 2017 [14]	In vitro experimental study	NIH Quality Assessment Tool for Before–After Studies Without Control Group	Low	Standardized organ bath procedures, reproducible experimental conditions, and systematic concentration–response testing minimized methodological variability and confounding.
Tanaka-Totoribe et al., 2005 [15]	Ex vivo vascular study	NIH Quality Assessment Tool for Before–After Studies Without Control Group	Low	Controlled ex vivo methodology with clearly described tissue handling and precise dose–response assessment reduced procedural and measurement bias.
Tabel et al., 2002 [16]	Randomized controlled trial	RoB-2	Low	Adequate randomization and allocation concealment were reported. Outcomes were objective, data were complete, and no selective reporting or protocol deviations were identified.
Yin et al., 2023 [17]	Ex vivo and molecular analysis	NIH Quality Assessment Tool for Before–After Studies Without Control Group	Low	Robust laboratory procedures, standardized tension recording, and molecular validation (Cav1.2/Cav3.1 expression) ensured low measurement and procedural bias.
Akter et al., 2024 [18]	Non-randomized clinical study	ROBINS-I	Moderate	Non-random allocation introduced possible confounding. Baseline characteristics were provided, but not all confounders were fully controlled. Objective outcome measures minimized detection and attrition bias.

Discussion

This review systematically evaluated the comparative efficacy and safety of vasodilators used to prevent internal thoracic artery (ITA) spasm in coronary artery bypass grafting (CABG), integrating evidence from clinical, in vitro, and ex vivo studies. The primary objective was to compare vasodilatory performance, while secondary objectives included assessing safety, onset times, mechanisms of action, and optimal delivery strategies. Across the six included studies, glyceryl trinitrate-verapamil (GV) solution, papaverine, systemic diltiazem, and efonidipine were the main agents evaluated, each differing in pharmacologic pathways and administration methods.

Clinical studies consistently favored the GV solution. He et al. (1994) [13] reported approximately a 95% increase in free flow with intraluminal or topical GV compared with 53% in controls, while Akter et al. (2024) [18] found higher free flow in patients receiving GV versus papaverine during off pump CABG, with no deaths in the GV group compared to two in the papaverine group. In vitro experiments demonstrated that GV achieved complete relaxation faster than papaverine (onset 2.9 vs 8.1 minutes) and with higher potency (pIC50 6.54 vs 4.58) [14]. Papaverine alone blocked vasoconstriction at 100 µM in ex vivo ITA segments, confirming its efficacy [15]. Systemic diltiazem significantly improved first and second free-flow measurements compared with systemic nitroglycerin [16], and efonidipine induced dose-dependent relaxation while downregulating Cav1.2 and Cav3.1 channels in ex vivo studies [17].

The mechanisms of action and delivery routes of vasodilators influenced their efficacy. Papaverine primarily inhibits phosphodiesterase, thereby increasing intracellular levels of cyclic AMP and GMP and relaxing vascular smooth muscle [15]. GV combines nitric oxide-mediated vasodilation from glyceryl trinitrate with L-type calcium channel blockade from verapamil, producing rapid, potent relaxation [13,14,18]. Systemic diltiazem relaxes smooth muscle through L-type calcium channel inhibition [16], while efonidipine targets both L- and T-type channels, potentially enhancing distal arterial relaxation [17]. GV solution had the fastest onset, supporting topical or intraluminal use for immediate intraoperative spasm prevention, whereas systemic or ex vivo agents act more slowly but remain effective in maintaining vasodilation.

All vasodilators were generally well tolerated. GV solution showed no major complications and fewer adverse events than papaverine [13,18], systemic diltiazem was hemodynamically stable [16], and laboratory studies reported no safety concerns [14,15,17]. GV solution consistently provided rapid, potent, and safe vasodilation, with papaverine as an established but slower alternative. Systemic calcium channel blockers like diltiazem serve as adjuncts, while efonidipine is promising mechanistically but requires clinical validation. While our review focuses on pharmacologic vasodilators for ITA spasm prevention, recent evidence suggests that surgical technique is equally important. Preservation of perivascular fat, minimal mechanical manipulation, and skeletonized harvesting of the ITA have been shown to reduce endothelial injury and lower spasm rates. Therefore, the efficacy of vasodilator solutions should be interpreted in the context of optimal ITA preparation, where both pharmacologic and surgical factors contribute to graft patency and intraoperative stability.

Limitations of this review include heterogeneous study designs, small sample sizes, varied vasodilator types and delivery methods, and limited clinical data for newer agents. The limited number of included studies reflects the stringent eligibility criteria applied to isolate mechanistic and directly comparable assessments of ITA vasoreactivity, rather than an absence of broader literature on ITA spasm management. While this review employed a rigorous, design-appropriate risk-of-bias assessment across all included studies, several limitations must be acknowledged. The total number of studies included was small, with modest sample sizes, which may limit the generalizability of the findings. In addition, some clinical trials carried a moderate risk of bias, primarily due to non-random allocation or incomplete reporting, potentially contributing to residual uncertainty in effect estimates. These factors highlight the need for larger, well-designed multicenter trials to confirm the comparative efficacy and safety of ITA vasodilators and to strengthen the evidence base for clinical decision making. Future research should focus on well-designed, multicenter trials comparing GV, papaverine, and novel calcium channel blockers, assessing optimal dosing, onset, safety, and long-term graft patency alongside mechanistic studies of ITA spasm.

## Conclusions

This systematic review compared the efficacy, safety, mechanisms, and optimal delivery strategies of vasodilators used to prevent internal thoracic artery (ITA) spasm during coronary artery bypass grafting (CABG) across clinical, in vitro, and ex vivo studies. Glyceryl trinitrate-verapamil (GV) solution consistently demonstrated the most rapid, potent, and reliable vasodilation, achieving superior graft-free flow improvement with an excellent safety profile, making it the most effective intraoperative agent for spasm prevention. Papaverine remained effective, particularly at higher concentrations, though with slower onset and greater variability compared with GV. Systemic diltiazem provided additional spasm reduction and was hemodynamically well tolerated, while the novel dual L- and T-type calcium channel blocker efonidipine showed promising mechanistic efficacy ex vivo but requires clinical validation. Direct topical or intraluminal application yielded the most consistent vasodilatory effects across agents, emphasizing the importance of delivery route. Overall, the GV solution offers the most robust and practical approach for optimizing ITA preparation, reducing perioperative spasm incidence, and improving graft flow during CABG, although further large-scale randomized trials are needed to refine dosing strategies, evaluate long-term outcomes, and confirm the clinical applicability of emerging agents such as efonidipine.
